# Adjuvant visible incoherent polarised phototherapy in chronic skin defects: a single-arm prospective observational cohort study

**DOI:** 10.1007/s10103-026-04933-1

**Published:** 2026-06-24

**Authors:** Michal Trnka, Katarína Zifčáková, Silvia Dulanská, Ján Pánik

**Affiliations:** 1 Faculty of Medicine, Institute of Medical Physics and Biophysics, Comenius University Bratislava, Bratislava, Slovakia; 2 Slovak Medical University, Bratislava, Slovakia

**Keywords:** Visible incoherent polarised light, Photobiomodulation, Chronic skin defect, Ulcus cruris, Wound healing, Adjuvant therapy

## Abstract

Chronic skin defects, particularly venous leg ulcers, impose a substantial clinical and socio-economic burden. This single-arm observational study was undertaken to describe the healing dynamics, pain outcomes, feasibility and tolerability of adjuvant visible incoherent polarised (VIP) phototherapy in patients with chronic skin defects who were managed with full standard care, and to examine whether outcomes varied with the principal clinical problem and treatment duration.

A prospective, single-arm cohort of 50 patients (38 females, 12 males; mean age 69.4 years) was observed. Adjuvant VIP phototherapy (626 nm; 2 mW/cm^2^) was applied alongside the standard conservative and surgical wound care received by all patients, for 10–20 min per field, two to three times daily; the continuous emission mode was changed to a pulsed mode (5 Hz) after 7–10 days. Wound area was assessed planimetrically in a representative case, and pain on a 0–10 numeric scale. No sham-irradiated control group was included.

Positive healing dynamics were recorded in 92% of the cohort and complete defect closure in 52% (*n* = 26). In the chronic-pain subgroup (*n* = 17), pain was reduced in 12 patients and resolved in 5. Improvement was associated with the principal clinical problem (χ^2^ = 10.406; *df* = 3; *p* = 0.015) and with therapy duration (χ^2^ = 8.630; *df* = 3; *p* = 0.035). Two patients reported mild, transient local reactions; no severe adverse events occurred.

In this uncontrolled cohort, adjuvant VIP phototherapy was feasible, well tolerated and accompanied by favourable healing and pain outcomes. Because no control group was studied, these findings cannot be attributed to the light itself and are hypothesis-generating; randomised, sham-controlled trials are required.

## Introduction

Chronic skin defects, and venous leg ulcers (ulcus cruris venosum) in particular, represent a severe and globally prevalent epidemiological problem that imposes a considerable socio-economic burden on healthcare systems [1–3]. The aetiology of these lesions is multifactorial, but the majority of leg ulcers are of venous origin and are associated with chronic venous insufficiency [4]. Pathophysiologically, the condition is characterised by sustained venous hypertension, which gives rise to microcirculatory disturbance and local tissue hypoxia [5]. The resulting disruption of local homeostasis and the exhaustion of reparative mechanisms tend to produce prolonged inflammation, so that the wound fails to progress through the physiological phases of healing within a predictable timeframe [6].

Conservative management, comprising surgical or enzymatic debridement, moist wound healing protocols and, for venous disease, targeted compression therapy, remains the clinical standard of care [7–9]. These interventions are, however, frequently constrained by prolonged healing trajectories, high recurrence rates and limited patient adherence, the latter partly attributable to therapy-associated pain and discomfort [9]. There is consequently continued interest in non-invasive adjuvant strategies that might support tissue repair and reduce pain when added to standard care; low-level laser therapy, for example, has been evaluated as an adjunct to conventional treatment in diabetic foot ulcers [3, 10].

Photobiomodulation (PBM), historically termed low-level laser therapy or, in the context of polarised light sources, visible incoherent polarised (VIP) phototherapy, has been investigated as an adjuvant modality for compromised tissue repair [10, 11]. VIP phototherapy employs low-energy, non-ionising radiation within the visible spectrum (predominantly red light around 626 nm) and is reported to exert photochemical effects on tissue without inducing thermal damage [12]. According to the established mechanistic literature (none of which was tested in the present study) the primary chromophore is held to be cytochrome c oxidase within the mitochondrial respiratory chain. Photon absorption is proposed to increase the mitochondrial membrane potential and adenosine triphosphate (ATP) synthesis, to modulate the inflammatory response, to enhance microcirculation, and to stimulate the proliferation and migration of fibroblasts and keratinocytes [13–15]. The response is widely described as biphasic, in accordance with the Arndt-Schulz principle, whereby low doses are stimulatory and excessive doses may be inhibitory [16].

In clinical use, VIP phototherapy is delivered in distinct operative modes. Continuous irradiation is reported to deliver energy rapidly and has been associated with vasodilation and with an analgesic effect attributed to stabilisation of nociceptor membranes [17]. A subsequent transition to a pulsed emission mode (for example 5 Hz) is frequently described as a means of limiting cellular habituation and sustaining responsiveness to the stimulus [18]. It should be emphasised that these mode-specific rationales are drawn from prior literature and from the device protocol, and were not the subject of measurement in the present study.

Despite this mechanistic interest, the clinical evidence base for VIP phototherapy in chronic skin defects remains limited and is dominated by heterogeneous protocols and small, frequently uncontrolled series, so that its real-world feasibility, tolerability and place within standard wound-care pathways are still poorly defined [10, 11]. Robust causal evidence can be provided only by randomised, sham-controlled trials, which remain scarce for this modality. Pragmatic observational data nonetheless retain value at this stage: they characterise how the intervention performs when added to routine care in an elderly, comorbid and aetiologically diverse population, document tolerability over the prolonged exposures that such wounds require, and generate the effect-size and feasibility estimates needed to design and power future controlled studies. The present report was undertaken in this spirit, as a descriptive, hypothesis-generating account rather than as a test of efficacy.

Although the biophysical rationale for PBM is well documented, its routine integration into wound-care algorithms requires further controlled clinical evaluation. Against this background, the present single-arm observational study was undertaken to describe, in a real-world cohort of patients with chronic skin defects managed with full standard care, the dynamics of healing and the modulation of pain during adjuvant VIP phototherapy (626 nm; 2 mW/cm^2^), and to examine whether the degree of improvement varied with the principal clinical problem and with the duration of therapy. Given the absence of a control group, the study was not designed to establish a causal effect of the light itself.

## Materials and methods

### Study design and reporting

A prospective, single-arm observational cohort study was conducted over a 12-month period at a university surgical clinic and a residential facility for seniors and social services. No control or sham-irradiation group was included, and no randomisation was performed. The study was therefore designed to characterise outcomes during adjuvant phototherapy added to standard care, not to establish a causal effect of the light. As an observational study of an established adjuvant treatment delivered within routine clinical care, rather than a randomised clinical trial assigning participants to interventions, the study was not registered in a clinical-trials registry. The report follows the STROBE recommendations for observational studies.

### Participants

The cohort comprised 50 patients (38 females, 12 males) with a mean age of 69.4 years (range 20–94 years). Patients were enrolled in collaboration with the treating physicians of each facility. Inclusion required the presence of a chronic skin defect, an age above 18 years and written informed consent. Exclusion criteria were malignant disease localised to the wound area, documented photosensitivity, and active deep vein thrombosis without adequate anticoagulation. The principal clinical problem was classified into four categories (i) venous leg ulcer, (ii) chronic pain, (iii) mixed-aetiology wounds and (iv) other defects; and the resulting distribution is summarised in Table 1. It should be made explicit that “chronic pain” denotes patients in whom pain, rather than a measurable defect, was the dominant presenting problem (in these patients the principal outcome assessed was pain rather than wound area). This aetiological and symptomatic heterogeneity is acknowledged as a limitation and is considered further in the *Discussion*.

**Table 1:** Baseline demographic and clinical characteristics of the patient cohort

Parameter	Subcategory	Number of patients (*n*)	Percentage (%)
Gender	Females	38	76
	Males	12	24
Age interval (years)	< 44	3	6
	45–55	7	14
	56–65	8	16
	66–75	8	16
	76–85	16	32
	> 86	8	16
Principal clinical problem	Venous leg ulcer	17	34
	Chronic pain	17	34
	Mixed aetiology wounds	6	12
	Other defects	10	20

### Standard wound care

Standard wound-care protocols were applied to all participants throughout the study, tailored to the aetiology of the lesion. This care comprised regular surgical or enzymatic debridement, antiseptic lavage (for example Ringer’s solution) and modern moist wound-healing dressings [19]. In patients with venous leg ulcers, appropriately fitted compression therapy was maintained [9]. Comorbidities were managed pharmacologically in accordance with standard clinical practice. Because every patient received this full standard-care regimen, all of which has independent therapeutic value, the design does not permit any added effect of phototherapy to be isolated.

### VIP phototherapy protocol and dosimetry

Adjuvant phototherapy was administered using a Biostimul light-emitting diode device (models BS 102 and BS 303; Biotherapy, Prague, Czech Republic), emitting visible incoherent polarised light at a characteristic wavelength of 626 nm. Pre-specified microclimatic conditions were maintained during exposure: ambient temperature 20–24 °C, relative humidity 60–65% and ambient illumination not exceeding 50 lx. The light was applied perpendicularly to the wound surface and the immediate peri-ulcer area from a constant distance of 1–1.5 cm. For larger defects, the light was applied statically and sequentially to adjacent, non-overlapping fields until the whole wound had been irradiated. Each session lasted 10–20 min per field and was repeated two to three times daily. The total duration of therapy was individualised according to the healing trajectory and ranged from 5 to 349 days. Selected patients were trained to continue self-application at home.

The protocol was biphasic. For the first 7–10 days a continuous emission mode was used. The device was then switched to a pulsed mode (5 Hz). As stated in the *Introduction*, the rationale for this sequence is drawn from the device protocol and prior literature and was not tested here.

Dosimetric parameters were derived from the manufacturer’s technical documentation. The irradiance (*I*) of the high-luminosity diodes was 2 mW/cm^2^ in continuous mode (within the device’s specified output range) over an effective static field of approximately 18–20 cm^2^. For a standard 15-min session (*t* = 900 s), the single-session fluence (*F*) was calculated as *F* = *I* · *t* = 2 mW/cm^2^ · 900 s = 1 800 mJ/cm^2^, that is 1.8 J/cm^2^. Although fluences of 2–6 J/cm^2^ are frequently recommended for deeper wounds [20], the present protocol delivered a lower single-session dose and relied on frequent, repeated daily applications. The cumulative dose was therefore not quantified, which is acknowledged as a limitation.

### Outcomes and assessment

The outcomes were defined a priori as follows. Overall clinical outcome was classified into four mutually exclusive categories: (i) complete closure/resolution, (ii) partial improvement, (iii) persistent/unchanged and (iv) deteriorated. “Positive healing dynamics” was defined as a composite of complete closure or partial improvement (that is, any documented favourable change, comprising granulation onset, exudate reduction and/or measurable area reduction), as opposed to persistent or deteriorated status. This is a non-standardised composite and is interpreted accordingly.

The overall clinical outcome was assessed for the whole cohort using the four-category classification defined above. In addition, quantitative wound-area measurement was performed for a representative case using a standardised clinical photo documentation protocol. Images were acquired with a digital single-lens-reflex camera fitted with a fixed-focal-length macro lens and a dedicated flash, positioned perpendicular to the wound at a standardised, fixed working distance under controlled illumination and against a flat, matte background. A rigid metric reference scale and a calibrated colour chart were included within every frame to permit dimensional calibration and colour correction; flexible or paper scales were not used. Each image was assigned a unique identifier and archived with its acquisition metadata, and an unmodified master copy was retained alongside the working copy. Wound area was then derived from the calibrated photographs in ImageJ (version 1.54; National Institutes of Health, USA) by delineating the wound margin as the region of interest [21]. Because calibrated quantitative area was obtained for the representative case rather than for the whole cohort, and was not read blind to timepoint, the area measurement is presented as an illustrative single-case observation and is interpreted accordingly.

Secondary outcomes were the time to complete closure (defined as full epithelialisation with absence of exudate), visual assessment of granulation-tissue quality, and pain. Pain intensity was rated subjectively on a 0–10 numeric scale [22]. Adverse events were recorded throughout.

### Statistical analysis

Associations between the degree of improvement and categorical predictors were examined using the Pearson chi-square (χ^2^) test, with significance set at *p* < 0.05. For these tests, the degree of improvement was dichotomised as complete healing/resolution versus partial (not fully healed). Two associations were tested: (i) improvement versus the principal clinical problem (four groups; 4 × 2 contingency table; *df* = 3) and (ii) improvement versus therapy-duration band (four duration bands: ≤8, 8–22, 23–38 and > 38 days; 4 × 2 contingency table; *df* = 3). Because the minimum expected cell frequency was below five in both tables, these associations are interpreted cautiously and regarded as exploratory. Quantitative wound area was available only for a representative illustrative case and not as a cohort-wide longitudinal dataset and no inferential test was therefore applied to wound area, which is reported descriptively for that single case.

## Results

### Overall clinical outcome

Positive healing dynamics, defined as complete closure or partial improvement, were recorded in 92% of the cohort (46/50). Complete defect closure or full resolution of the principal problem was documented in 52% (*n* = 26), partial improvement in 40% (*n* = 20) and a persistent, unchanged status in 8% (*n* = 4); no patient deteriorated. The macroscopic clinical outcomes are summarised in Table 2.

The temporal response varied considerably between patients. Early favourable indices (onset of granulation and reduced exudate) were noted within 5–8 days in some patients, whereas the longest individual times to complete closure of extensive defects were 225 and 349 days.

**Table 2:** Macroscopic clinical outcome following adjuvant VIP phototherapy (*n* = 50)

Clinical outcome	Number of patients (*n*)	Percentage (%)
Complete closure/resolved	26	52
Partial improvement	20	40
Persistent/unchanged	4	8
Deteriorated	0	0
Total	50	100

### Defect area

**Fig. 1:**
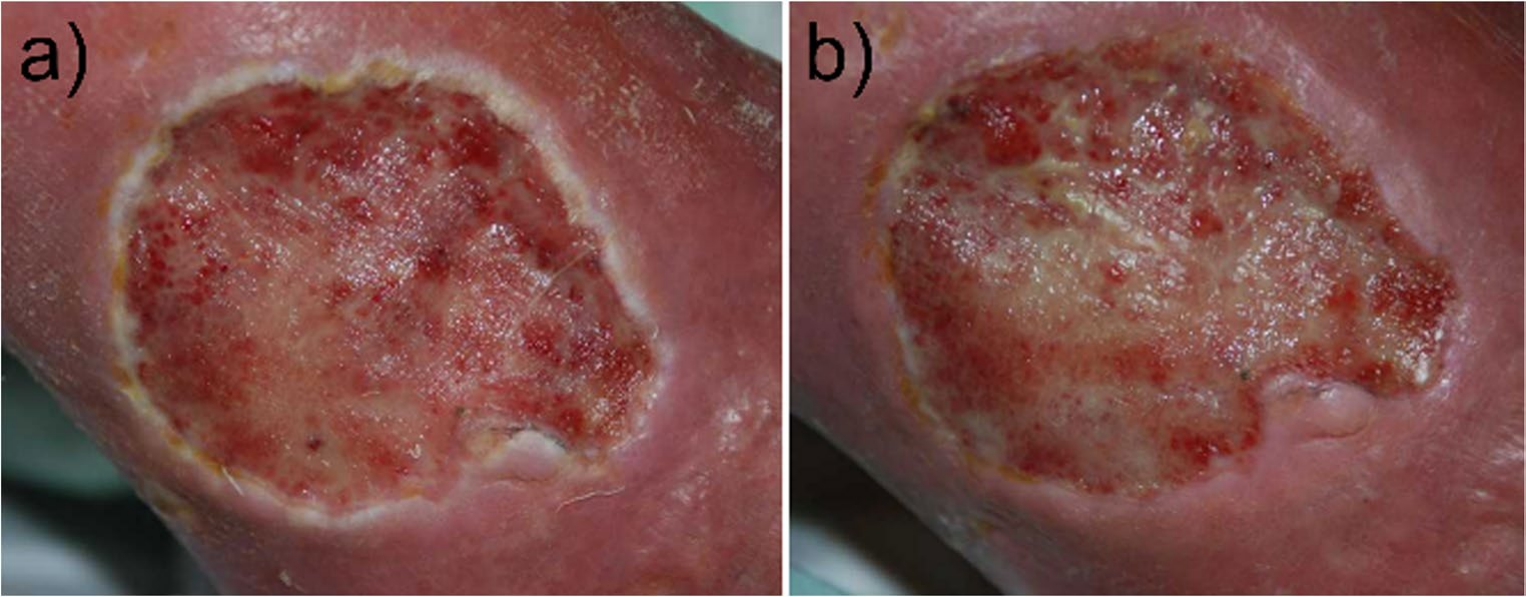
Macroscopic appearance of a representative venous leg ulcer during adjuvant VIP phototherapy. **a** Baseline, before phototherapy, with prominent fibrinous slough. **b** After 30 days of adjuvant phototherapy, with granulation tissue and marginal re-epithelialisation. This single case is shown for illustration only and does not support cohort-level inference

**Fig. 2:**
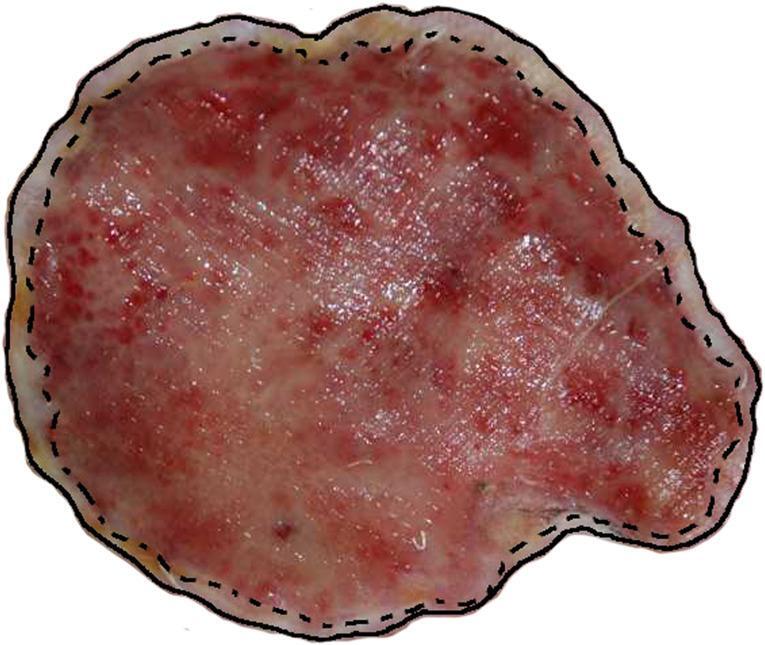
Planimetric delineation of a representative chronic skin defect. The dashed line marks the wound margin used as the region of interest for area measurement from the digital photograph. The image is shown for illustration of the measurement method only and does not represent cohort-level data

Quantitative wound-area assessment is illustrated for a single representative patient with a venous leg ulcer. In this case, the calibrated planimetric area decreased from 48.0 cm^2^ at baseline to 40.0 cm^2^ after 30 days, a reduction of approximately 17% (8.0 cm^2^). The macroscopic appearance of this wound before and after is shown in Fig. 1, and the corresponding planimetric delineation of the wound margin is shown in Fig. 2. As only a single case was measured quantitatively, this observation is illustrative and is not presented as a cohort-level result. The cohort outcomes are the categorical findings reported above.

In the venous leg ulcer subgroup (*n* = 17), complete epithelialisation was achieved in 11 patients (64.7%) and partial resolution in the remaining 6 (35.3%). Among patients with available timing data, the mean time to the first signs of partial healing was 10.33 days (SD 2.34) and the mean time to complete healing was 43.78 days (SD 25.01). The wide dispersion of the latter reflects the individual variability of the healing trajectories and is consistent with the broad overall range of treatment durations.

### Pain and tolerability

Within the subgroup in whom chronic pain was the principal problem (*n* = 17), pain intensity was reduced in 12 patients and resolved completely in a further 5. This improvement was reported in conjunction with subjectively better sleep. The intervention was well tolerated. No severe or systemic adverse events occurred. Mild, transient local reactions, a subjective sensation of warmth or transient erythema at the irradiation site, were reported by 2 patients. Both resolved spontaneously and required no interruption or modification of the protocol.

### Associations between improvement, diagnosis and therapy duration

For the analyses of association, outcome was dichotomised into complete healing/resolution versus partial response. On this ordinal classification, 29 patients (58%) were categorised as having achieved complete healing and 21 (42%) a partial response. This dichotomy is not interchangeable with the four-category macroscopic outcome in Table 2. The latter reflects categorical wound status at the common observation point (26 complete closures), whereas the dichotomy reflects the final status assigned across individualised follow-up periods of 5–349 days. The difference of three patients is attributable to the use of two distinct assessment instruments applied at different points, rather than to a discrepancy within a single variable.

The degree of improvement was associated with the principal clinical problem (Pearson χ^2^ = 10.406; *df* = 3; *p* = 0.015; Table 3) and with the duration of therapy (χ^2^ = 8.630; *df* = 3; *p* = 0.035; Table 4). Because the minimum expected cell frequency was below five in both tables, these associations are interpreted with caution and regarded as exploratory. Complete healing was least frequent in the chronic-pain subgroup, which showed predominantly partial responses, and more frequent in the wound-based groups. Longer treatment durations were associated with a higher proportion of complete responses.

**Table 3:** Association between the degree of improvement and the principal clinical problem (Pearson χ^2^ = 10.406; *df* = 3; *p* = 0.015)

Principal clinical problem	Partial response (*n*)	Complete healing (*n*)	Total (*n*)
Venous leg ulcer	6	11	17
Chronic pain	12	5	17
Mixed-aetiology wounds	2	4	6
Other defects	1	9	10
Total	21	29	50

**Table 4:** Association between the degree of improvement and the duration of therapy (Pearson χ^*2*^ *=* 8.630; *df* = 3; *p* = 0.035)

Duration of therapy	Partial response (*n*)	Complete healing (*n*)	Total (*n*)
≤ 8 days	8	10	18
8–22 days	12	8	20
23–38 days	1	5	6
> 38 days	0	6	6
Total	21	29	50

## Discussion

### Principal findings

This single-arm observational study describes the outcomes of patients with chronic skin defects during adjuvant VIP phototherapy added to full standard wound care. Positive healing dynamics were recorded in 92% of the cohort and complete closure in 52%, and pain was reduced or resolved in the majority of the chronic-pain subgroup. Quantitative wound-area reduction was documented in a single illustrative case (Figs. 1 and 2) and is not a cohort-level finding. The intervention was well tolerated, with only mild, transient local reactions and no severe adverse events. Because the study was uncontrolled and every patient concurrently received debridement, antiseptic lavage, moist-wound dressings and, where indicated, compression (each of which has independent therapeutic value) these outcomes are fully compatible with the expected course of standard care alone, and no added effect of the light can be inferred from the present design. The findings are therefore interpreted as feasibility, tolerability and hypothesis-generating observations rather than as evidence of efficacy.

### Mechanistic context

A biophysical rationale for photobiomodulation is well described in the prior literature and is summarised here only as background. None of the processes named below was measured in this study, and they are not offered as an explanation of the present clinical observations. Photon absorption by cytochrome c oxidase is proposed to raise the mitochondrial membrane potential and ATP synthesis, to modulate inflammation, and to influence microcirculation, with a dose-response relationship widely held to be biphasic in accordance with the Arndt-Schulz principle [13, 16, 23]. The mode-specific rationale (continuous irradiation for early energy delivery and a subsequent pulsed mode to limit cellular habituation) derives likewise from the device protocol and earlier reports [24, 25]. Because no ATP, perfusion, nitric-oxide, temperature or histological readout was obtained, these mechanisms cannot be used to validate the clinical findings, which must stand on the study design alone.

### Pain modulation

In the chronic-pain subgroup, pain was reduced in 12 of 17 patients and resolved in 5, in association with subjectively improved sleep. Mechanisms proposed in the literature for PBM-related analgesia include improved local perfusion, accelerated clearance of algogenic mediators and stabilisation of nociceptive C-fibres [26]. As above, none was assessed here, and the present observation is uncontrolled and based on subjective rating. It is reported only as a tolerability-related secondary observation.

### Comparison with the literature

The favourable healing and tolerability observed here are broadly consistent with reports of light and laser-based approaches in cutaneous repair, although these vary widely in source, wavelength, dose and design and are not directly comparable with the present protocol. Controlled data are more informative. A randomised controlled trial of polarised light in diabetic foot ulcers reported benefit on healing and wound microbiota [11], and polarised polychromatic light has been studied in pain-related recovery [12]. Light-based interventions have also been evaluated in other cutaneous indications, such as a non-ablative fractional 1 340 nm laser for stretch marks, which illustrates both the breadth of the field and the importance of indication-specific dosing and controlled evaluation [27]. Set against this background, the contribution of the present study is limited and pragmatic. It documents real-world feasibility and tolerability in an elderly, comorbid and heterogeneous population, rather than adding to the controlled evidence base [10].

### Strengths and limitations

The main strength is the pragmatic, real-world setting and the prolonged follow-up in an elderly, comorbid population in whom controlled trials are scarce. The limitations are substantial and constrain interpretation. First and most importantly, the single-arm design without a sham-irradiation control, layered on full standard care, does not permit any effect to be attributed to the light. Second, the cohort was aetiologically heterogeneous, and the chronic-pain subgroup was defined by a symptom rather than by a measurable defect, so subgroup denominators are not directly comparable. Third, although wound photography followed a standardised, calibrated protocol, quantitative area in absolute units was obtained for a representative case only and not for the whole cohort, and the cohort-level outcome was therefore categorical rather than a continuous area change. The single-case area measurement is illustrative and was not read blind to timepoint. Fourth, the chi-square associations rested on contingency tables with expected cell frequencies below five and should be regarded as exploratory. Fifth, pain was rated subjectively and the cumulative delivered dose was not quantified. The wide range of treatment durations (5–349 days) further reflects this heterogeneity.

### Future directions

The principal need is for randomised, sham-controlled trials with aetiological stratification, cohort-wide quantitative planimetry with blinded, timepoint-masked reading and pre-specified denominators (for example, percentage area reduction analysed per patient with appropriate repeated-measures methods), and accounting for attrition. Dosimetric studies mapping the effective optical window for specific wound types, and evaluation of combined visible and near-infrared wavelengths, would help define whether, and at what dose, any genuine adjuvant effect exists.

## Conclusion

In this single-arm observational cohort of patients with chronic skin defects, adjuvant VIP phototherapy at 626 nm, added to full standard wound care, was feasible and well tolerated across prolonged treatment exposures, with no severe adverse events. Favourable healing dynamics were recorded in 92% of the cohort and complete defect closure in 52%, and pain was reduced or resolved in most of the chronic-pain subgroup. A reduction in wound area was additionally documented in a single illustrative case. The degree of improvement was associated with the principal clinical problem and with the duration of therapy, although these associations were exploratory. Because the study was uncontrolled and all patients concurrently received standard care of independent therapeutic value, these outcomes cannot be attributed to the light itself and are consistent with the expected course of standard care alone. The results should therefore be regarded as hypothesis-generating evidence of feasibility and tolerability rather than of efficacy. Randomised, sham-controlled trials with standardised, blinded planimetry and pre-specified denominators are required before adjuvant VIP phototherapy can be recommended for routine integration into wound-care algorithms.

## Data Availability

The data that support the findings of this study are available from the first author upon a reasonable request. All relevant data generated or analysed during this study are included in this manuscript or available at Zenodo public repositories; access to additional information can be granted upon reasonable request to the authors.
